# Research gaps and unmet challenges to study the impact of chemicals on neurodegenerative diseases

**DOI:** 10.1002/alz.087446

**Published:** 2025-01-09

**Authors:** Cécilia Samieri, Sophie Lefèvre‐Arbogast, Jade Chaker, Fabien Mercier, Robert Barouki, Xavier Coumoul, Gary W Miller, Arthur David

**Affiliations:** ^1^ Bordeaux Population Health Research Center, Inserm U1219, University of Bordeaux, Bordeaux France; ^2^ Univ Rennes, Inserm, EHESP, Irset (Institut de Recherche en Santé, Environnement et Travail), Rennes, NA France; ^3^ Université Paris Cité, T3S, INSERM UMR‐S 1124, Paris, NA France; ^4^ Mailman School of Public Health, Columbia University, New York, NY USA

## Abstract

Chemicals are ubiquitous in modern life. More than 100,000 chemicals are currently used worldwide, and the production continues to increase. Measuring with accuracy all the components of the chemical exposome represents a tremendous challenge which has been only very partly met so far, and gaps in science remain multiple. For example, there is a need for a comprehensive understanding of the chemical exposome’s impact on the brain, emphasizing the integration of various exposure routes and an expanded list of emerging concern chemicals. Biomonitoring, a relatively recent approach, has begun linking internal exposure to neurological impacts, shedding light on the potential neurotoxicity of non‐persistent pesticides like neonicotinoids and pyrethroids. Research should explore overlooked ubiquitous chemicals, such as flame retardants, fluorosurfactants, plasticizers, and food additives, and overlooked pathways such as neurovascular dysfunction or the impact on non‐neuronal cells.

Furthermore, analytical challenges posed by exposomic studies are specifically important, since the chemical space is highly variable, dynamic and diverse in concentration levels (with ultra‐trace substances). Chemical exposures are multiple and potential additive or synergistic effects of chemicals, within mixtures, likely underlie the impact of the chemical exposome on the brain. Capturing the depth and breadth of that chemical exposome necessitates cutting‐edge molecular markers. The development of high throughput omics‐based molecular approaches has allowed simultaneous analysis of thousands of individual biological entities at a large scale – a technological revolution which has enabled the development of exposomics. High‐resolution mass spectrometry (HRMS)‐based methods applied to various matrices, allows to uncover unknown elements of the internal chemical exposome associated with neurodegenerative diseases.

This presentation will discuss all the promises that new technologies combined with omics‐based methodologies could provide to uncover unknown chemical signatures associated to neurodegenerative diseases.

